# Supplementary material for: Multi-Center National Study of Genotype–Phenotype Correlation and Clinical Characteristics in Children and Young Adults with Friedreich’s Ataxia from Serbia
Source: Biomedicines. 2025 Oct 28;13(11):2646. doi: 10.3390/biomedicines13112646 (PMC12650222; doi:10.3390/biomedicines13112646)
Supplement: Supplementary file 1 [file biomedicines-13-02646-s001.zip › biomedicines-3892101-supplementary.pdf]

Table S1. Comparison of GAA1 length, GAA2 length, age at onset, and disease duration between phenotype subgroups defined by the presence or absence of respective clinical features. For comparison of group means Wilcoxon rank sum was used.

Clinical feature	GAA1		GAA2		Age at onset		Disease duration	
	Mean length	P-value	Mean length	P-value	Mean age	P-value	Mean duration	P-value
**Cardiomyopaty**								
Present	823.7 (n=18)	0.188	1025 (n=18)	0.986	8.46 (n=22)	0.084	8.31 (n=22)	0.387
Absent	778.3 (n=7)		1035 (n=7)		10.86 (n=7)		7.36 (n=7)	
**Dysarthria**								
Present	817.7 (n=14)	0.231	1002 (n=14)	0.396	8.27 (n=16)	0.320	9.96 (n=16)	**0.003**
Absent	790.8 (n=12)		1043 (n=12)		8.83 (n=14)		5.64 (n=14)	
**Extensor plantar response**								
Present	825.5 (n=20)	**0.024**	1020 (n=20)	0.613	8.7 (n=20)	0.566	8.18 (n=20)	0.160
Absent	702.6 (n=5)		962.2 (n=5)		8.17 (n=6)		7.74 (n=6)	
**Vibration sense**								
Present	801.5 (n=24)	0.886	1011 (n=24)	**0.025**	8.93 (n=28)	0.609	8.08 (n=28)	0.706
Absent	850.5 (n=2)		962.2 (n=2)		10 (n=2)		6.12 (n=2)	
**Pes cavus**								
Present	815.7 (n=20)		1039 (n=20)		9.6 (n=23)		8.37 (n=23)	
Absent	759 (n=4)	0.273	1020 (n=4)	0.619	9.06 (n=5)	0.888	4.93 (n=5)	**0.031**
**Nystagmus**								
Present	723.7 (n=9)		1012 (n=9)		9.4 (n=10)		9.68 (n=10)	
Absent	840.8 (n=17)	0.396	1047 (n=17)	0.533	8.8 (n=20)	0.590	7.08 (n=20)	**0.036**
**Upper limb Areflexia**								
Present	812.7 (n=24)	0.301	1027 (n=24)	0.492	9.07 (n=27)	0.528	8.37 (n=27)	**0.015**
Absent	716.5 (n=2)		085 (n=2)		8.33 (n=3)		4.16 (n=3)	
**ECG abnormality**								
Present	806.4 (n=20)	0.354	1033 (n=20)	0.348	8.91 (n=22)	0.554	8.42 (n=22)	**0.036**
Absent	737 (n=3)		984.7 (n=3)		10.4 (n=5)		4.77 (n=5)	
**Loss of ambulation**								
Yes	844.4 (n=7)	0.280	1065 (n=7)	0.311	7.75 (n=8)	0.208	10.3 (n=13)	**0.002**
No	790.5 (n=19)		1009 (n=19)		9.46 (n=22)		6.1 (n=22)	

Legend: GAA1 and GAA2: the number of GAA repeats in the smaller and the larger *FXN* allele, respectively; values in parenthesis represent number of patients in the corresponding subgroup; bold: statistically significant difference.
